# Exploring the Impact of Individual UVB Radiation Levels on Serum 25-Hydroxyvitamin D in Women Living in High Versus Low Latitudes: A Cross-Sectional Analysis from the D-SOL Study

**DOI:** 10.3390/nu12123805

**Published:** 2020-12-11

**Authors:** Marcela M. Mendes, Kathryn H. Hart, Susan A. Lanham-New, Patrícia B. Botelho

**Affiliations:** 1 Department of Nutritional Sciences, Faculty of Health and Medical Sciences, University of Surrey, Guildford GU2 7XH, UK; k.hart@surrey.ac.uk (K.H.H.); s.lanham-new@surrey.ac.uk (S.A.L.-N.); 2 Department of Nutrition, Faculty of Health Sciences, University of Brasília, Brasília 70910-900, Brazil; patriciaborges.nutri@gmail.com

**Keywords:** vitamin D, sunlight exposure, latitude, vitamin D intake

## Abstract

Vitamin D can be synthesized in the skin via sunlight exposure as well as ingested through diet. Vitamin D deficiency is currently a major global public health issue, with increasing prevalence in both low and high latitude locations. This cross-sectional analysis aimed to compare the intensity of individual Ultraviolet B radiation levels between women of the same ethnicity living in England and Brazil, respectively; and to investigate the association with circulating 25(OH)D concentrations. We analysed data from 135 Brazilian women (England, *n* = 56, 51° N; Brazil, *n* = 79, 16° S) recruited for the D-SOL study (Interaction between Vitamin **D** Supplementation and **S**unlight Exposure in Women Living in **O**pposite **L**atitudes). Serum 25(OH)D concentrations were analysed by high performance liquid chromatography tandem mass spectrometry (HPLC-MS), individual UVB radiation via UVB dosimeter badges and dietary intake via 4-day diet diaries. Anthropometric, skin phototype, sociodemographic and lifestyle patterns were also assessed. Mean serum 25(OH)D concentration of England residents was significantly lower than Brazil residents. Daily individual UVB radiation level showed a strong significant positive correlation with serum 25(OH)D concentrations. The required UVB radiation to achieve 75 nmol/L was 2.2 SED and 38.8% of the total variance in 25(OH)D concentrations was explained uniquely by daily individual UVB radiation, after controlling for the influence of age and body mass index. Thus, these results highlight the strong positive association between serum 25(OH)D concentrations and individual UVB radiation and the influence of different individual characteristics and behaviours. Collectively, these factors contribute to meaningful, country-specific, public health strategies and policies for the efficient prevention and treatment of vitamin D inadequacy.

## 1. Introduction

Prolonged and severe vitamin D deficiency can lead to rickets in children and osteomalacia/osteoporosis in adults [1,2,3]. Vitamin D is naturally present in very few foods and in small quantities. The main source of Vitamin D is considered to be casual exposure of the skin to the UVB portion of sunlight (290–350 nm), which converts the molecule 7-dehydrocholesterol naturally present in the epidermis, to pre-vitamin D [2,3,4,5].

Sunlight will need to travel through the atmosphere before reaching the skin to produce vitamin D. The availability of UVB radiation is determined mainly by the solar zenith angle, which is influenced by latitude, season and time of day. Therefore, the solar irradiation on the surface of the earth, and concomitantly the local UVB radiation, will depend on the verticalization of the solar zenith angle in each particular latitude [3,6]. In other words, populations living within the tropics, namely low latitude locations (e.g., South America), are exposed to substantially higher levels of solar UVB radiation throughout the year than those living in high latitude locations (i.e., Europe) [6]. For instance, the annual total horizontal irradiation in Surrey, UK (latitude 51° N) is approximately 1000 kWh/m^2^ [7] in comparison to the annual average of 5500 kWh/m^2^ in Goiás, Brazil (16° S) [8]. Nevertheless, recent reports of an increasing prevalence of low vitamin D concentrations in both low and high latitude locations show that vitamin D deficiency is rapidly becoming, if not already, a major global public health issue [9,10,11,12,13].

Advisory agencies (Government led or otherwise) have consistently highlighted the challenges in establishing reference values for adequate vitamin D recommendations, particularly due to the individual variation as well as the influence of external environmental factors [14,15,16]. The inappropriateness of direct comparison of data from studies conducted in different locations is mainly due to significant variations in results between different laboratories, different latitudes and different populations/ethnic groups—and therefore influencing factors, adding greatly to the difficulty in finding a global consensus. Moreover, very few studies to date have investigated the relationship between actual individual exposure to UVB radiation and serum 25(OH)D concentrations [6,17,18], with most data derived from in vitro or animal studies. Another important limitation to the current recommendations regarding optimal dietary intakes and sunlight exposure to maintain adequate levels, is that they are generally based on studies of Caucasian populations in high latitude countries, with limited robust data for other ethnicity and/or different geographical locations. Consequently, there is a substantial lack of evidence on the effect of individual sunlight exposure in low latitude countries and in their native non-Caucasian populations.

In order to develop effective vitamin D guidelines, we need to fully understand the actual impact of sunlight on 25(OH)D concentrations, based not only on UVB radiation availability (latitude) but also individual UVB radiation exposure, as well as the relative contribution of key influential factors, such as dietary intake, adiposity, skin pigmentation and lifestyle. Therefore, the aim of this cross-sectional analysis was to compare the intensity of individual UVB radiation levels between Brazilian adult women living in England and Brazilian adult women living in Brazil, and to investigate the association with circulating 25(OH)D concentrations.

## 2. Materials and Methods

This is a cross-sectional analysis of data collected by the D-SOL study (Interaction between vitamin D supplementation and Sunlight exposure in women living in Opposite Latitudes; registered at clinicaltrials.gov as NCT03318029). The study was approved by the University of Surrey (UEC/2016/009/FHMS) and Federal University of Goiás Ethics Committees and by the Brazilian National Ethics Committee (CONEP) (CAAE 62149516.9.0000.5083, CEP-UFG nº 2013222; CONPEP nº 1972029; respectively). All participants at commencement of the study provided written informed consent.

### 2.1. Study Location

The D-SOL study was conducted at the University of Surrey, Guildford, Surrey (51° N), England, from December 2016 to March 2017 (high latitude group) and at the University of Goiás, Goiás (16° S), Brazil, from June to September 2017 (low latitude group). Surrey has a temperate climate (throughout the year a maximum of ~25 °C and minimum of ~0 °C) with a summer mean temperature of 22 °C and winter mean temperature of 6 °C, whereas Goiás has a typical tropical savannah climate (throughout the year a maximum of ~36 °C and minimum of ~15 °C), with a summer mean temperature of 26 °C and winter mean temperature of 24 °C. The UV index never exceeds 8 in the UK (peaking towards the end of June), and in clear contrast, the minimum in Brazil is 8 in winter, reaching up to 14 during hotter months.

### 2.2. Study Design

Participants were selected if they were female of Brazilian nationality (born in Brazil and having at least one parent born in Brazil), aged 20–59 years. Exclusion criteria included: currently receiving treatment for medical conditions that are likely to affect vitamin D metabolism (osteoporosis therapy, anti-estrogens treatment, antiepileptic drugs, cancer treatment); taking supplements containing vitamin D (if the prospective participants agreed to stop vitamin D supplementation to join the study, a wash-out period of 8 weeks prior to commencing the trial was accepted), being pregnant or planning a pregnancy during the study period, being post-menopausal (defined as permanent cessation of menstruation) and living in the UK for less than 3 months at the commencement of the study (for England participants only).

Participants in England were recruited through advertisements distributed locally in Surrey and London. Brazilian institutions in the UK, such as the Brazilian Embassy in London and the Brazilian Researchers Association (ABEP-UK), agreed to circulate a recruitment letter to their contact lists. Participants in Brazil were also recruited locally form the residents of the city of Goiânia, Goiás. For both trials, recruitment via social media and online platforms was also used.

### 2.3. Anthropometric Measures

For measurement of weight participants were asked to remove shoes, socks and heavy coats before stepping on the scale (England: Tanita Body Composition Analyser MC-180MA, Tanita Coopertatives, Tokyo, Japan; Brazil: standard weighing scale, Balmak^®^). Waist circumference was measured with a non-extendable standard measure tape, at the narrowest point of the torso, to the nearest 0.1 cm. If this point could not be estimated, the level of the umbilicus was used as the reference point. Body Mass Index (BMI) was classified according to the World Health Organization as underweight (<18.5 kg/m^2^), normal weight (18.5–24.9 kg/m^2^), overweight (25.0–29.9 kg/m^2^) or obese (>30 kg/m^2^) [19].

### 2.4. Lifestyle and Sun Exposure

A lifestyle questionnaire, adapted for each country, was administered to assess for cultural and general lifestyle aspects. Dietary intake of participants was determined by 4 consecutive days of estimated diet diaries, commencing on a Sunday to ensure weekdays and weekend days were represented. Participants were trained by the research team on how to correctly complete the diary.

Participants were instructed to maintain their habitual sunlight exposure and sun protective measures (if part of their habitual routine) as well as their usual dietary intake for the duration of the study and were requested to report any significant changes to their habits or normal routine.

To determine individual exposure to ambient UVB radiation, participants were asked to wear individual polysulphone film badge dosimeters (provided by the University of Manchester, UK) on their outer clothing. Participants were instructed to wear their dosimeters around the upper shoulder/chest region from sunrise to sunset for a full week (7 consecutive days), starting the day after blood samples were taken. All dosimeters, for both the England and Brazil trials, were read at the University of Surrey, prior to and after use, with a Cecil Aquarius CE7200 Double Beam Spectrophotometer (which has a CV < 1%) at 330 nm, to detect change in absorbency [6]. The amount of UVB captured by each dosimeter badge was then translated to a standard erythematous dose (SED): 1 SED is equal to 100 J m^−2^ of erythemal (sun burning) UVB radiation. A measure of 3 SED is roughly equivalent to one minimal erythema dose [MED] in unacclimatized, sensitive white skin. An exposure of 5–8 SED will result in moderate sunburn and 10 SED or more can result in a painful, blistering sunburn, in unacclimatized, sensitive white skin [18].

### 2.5. Skin Pigmentation

Race and skin type were both self-reported via the lifestyle questionnaire. Race categories were based on the Brazilian ethno-race national demographic spectrum [20], which includes: White, Black, Brown/Mixed (“Pardo” in Portuguese), Indigenous and “Yellow” (Asian-descendent). Participants were asked to indicate which category they most identified with. Skin type was based on the Fitzpatrick validated classification for skin photo-types, which classifies the skin according to the ability of each skin type to tan under sun exposure and its sensitivity and tendency to turn red under solar radiation [21]. Participants were asked to choose one category only that best represented the effect of sunlight exposure on their skins. For the purpose of this study, Fitzpatrick’s photo-types were combined into skin type categories as follow: Type I (Always burns; Never tan; Very sensitive to the Sun) and II (Always burning; Very little tan; Sun sensitive) were classified as ‘white’; Type III (Burns moderately; Bronze moderately; Normal sensitivity to the Sun) and IV (Burns a little; Always tan; Normal sensitivity to the Sun) as ‘brown’; Type V (Rarely burns; Always tan; Not sensitive to the sun) and VI (Never burn; Totally pigmented; Insensitive to the Sun) as ‘black’.

### 2.6. Laboratory Analysis

An overnight fasted (8 h) blood sample was collected by venipuncture by trained phlebotomists in both centres. For serum, the collected blood samples were left to clot for 1 h at room temperature followed by centrifugation at 3000 *g* for 10 min at 4 °C (England trial: Sigma 3–16 K Centrifuge, SciQuip, Shropshire, UK; Brazil trial: Eppendorf™ 5702R Centrifuge, UK). Processed serum samples were distributed into aliquots and stored at −80 °C at the University of Surrey, prior to analysis. Samples collected in Brazil followed the exact same procedures and were temporarily stored at −80 °C at the University of Goiás. The samples were then sent by air to the UK to be stored at the University of Surrey, prior to analysis.

All samples, from both countries, were analysed for 25(OH)D, serum calcium and serum albumin at Imperial College London. Serum 25(OH)D concentrations were determined by HPLC-MS/MS method on a Waters Acuity TQD using a PFP column following supported liquid extraction (SLE). Laboratory intra- and interassay CVs were 5.6% and 7.8%, respectively. Serum calcium was measured by using an endpoint spectrophotometric reaction based on the o-cresolphthalein complexone methodology, and serum albumin was measured by using an endpoint spectrophotometric reaction based on the bromocresol green solution dye binding methodology. Serum calcium concentrations were adjusted for albumin concentrations.

Due to the lack of global consensus as to the definition of vitamin D status, for the purpose of this study vitamin D deficiency was defined as 25(OH)D concentrations below 25 nmol/L, as suggested by the UK Scientific Advisory Committee on Nutrition [14]; insufficiency as concentrations between 25–49.9 nmol/L and adequacy between 50–74.9 nmol/L, as recommended by the US Institute of Medicine [15]; and optimal levels as concentrations above 75 nmol/L, as proposed by the US Endocrine Society [16].

### 2.7. Statistical Analysis

Statistical analysis of the data was undertaken using SPSS software for Windows (version 26.0, 2019; IBM Corp, Armonk, NY, USA). Data were tested for normality using the Kolmogorov-Smirnov test. Non-normally distributed variables were log transformed and reported in the original scale. Non-parametric tests were used when log transforming did not normalize the data. For categorical variables, frequency and percentage were reported. The distribution of skin type and BMI classification were compared between countries using chi squared tests.

Mean circulating serum 25(OH)D concentrations were compared between different aspects of lifestyle, individual characteristics and individual UVB radiation levels using independent t-tests, or Mann-Whitney U tests for non-normally distributed data; or one-way ANOVA with post-hoc Tukey tests, or Kruskal-Wallis for non-normally distributed data. Standard linear regression models were run to investigate the predictive ability of individual daily sunlight exposure on circulating serum 25(OH)D concentrations.

A *p* value of <0.05 was considered significant.

## 3. Results

**Figure 1:**
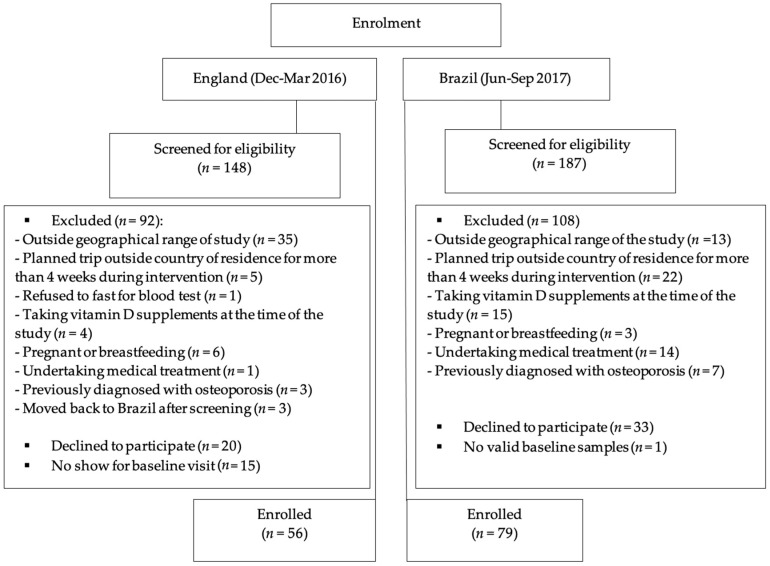
Flow diagram of participant enrolment.

Of the 335 participants enrolled for the D-SOL study after the screening process, 135 participants were included in this cross-sectional analysis (*n* = 56 in England and 79 in Brazil). Reasons for exclusion at screening are detailed in Figure 1. In the Brazil cohort, one participant did not have valid laboratory results and was therefore excluded from the database as previously described in Mendes et al. (2020) [22]. In the England cohort, five participants were post-menopausal. There were no differences between analyses including or excluding the five post-menopausal women, and therefore, only analyses including these participants are reported here.

### 3.1. Participant Characteristics

Participant characteristics, specifically age, weight, BMI, vitamin D and calcium intake and 25(OH)D, PTH and calcium serum concentrations, have been previously published in Mendes et al., 2019 [22]. Brazilian women living in England were older, heavier and had a greater waist circumference than those living in Brazil (*p* < 0.01). There were no significant differences between Brazilian women living in England and in Brazil for BMI classification distributions although, in line with the weight data, the mean BMI was significantly greater for Brazilian women residing in England.

**Figure 2:**
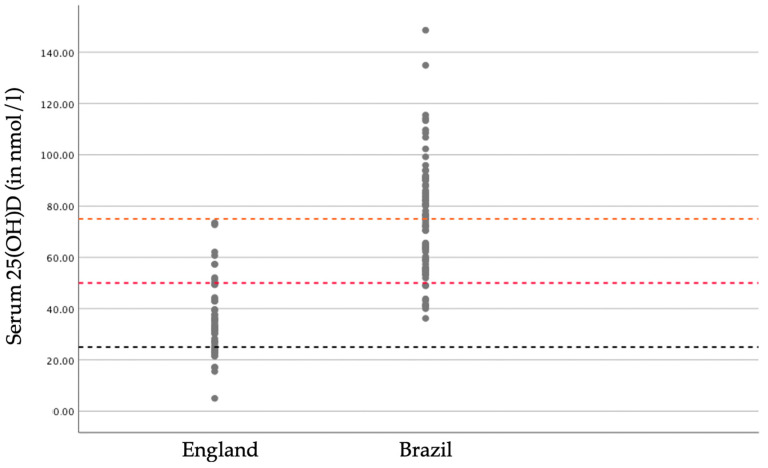
Serum 25(OH)D concentrations for women living in England (*n* = 56) and women living in Brazil (*n* = 79). Hashed lines represent thresholds of 25 (deficiency), 50 (insufficiency) and 75 (optimal) nmol/L.

Figure 2 shows the difference in serum 25(OH)D concentrations between the two countries, with concentrations ranging from 5.0 to 73.5 nmol/L for participants living in England and from 36.2 to 148.6 nmol/L for those living in Brazil. Mean serum 25(OH)D concentration of England residents was significantly lower than Brazil residents (36.0 ± 14.9 nmol/L and 75.0 ± 22.1 nmol/L, respectively *p* < 0.001), as previously published [22]. The statistical significance remained after controlling for daily UVB radiation level, age, BMI and waist circumference (ANCOVA, *p* < 0.001). Only participants living in Brazil had serum 25(OH)D concentrations above 100 nmol/L (*n* = 3), of which two women had concentrations above 130 nmol/L (134.9 and 148.6 nmol/L). There were no significant differences in serum albumin-corrected calcium concentrations between England and Brazil residents (2.3 ± 0.07 and 2.2 ± 0.06 mmol/L, respectively, *p* = 0.066), with all participants having concentrations within the normal range of 2.1–2.6 mmol/L.

The proportions of women with deficient and insufficient levels were significantly higher in those living in England than in Brazil (*p* < 0.001). Amongst women living in England 25% had serum 25(OH)D concentrations below 25 nmol/L, while there were no participants with concentrations below this threshold living in Brazil. There were no participants living in England with concentrations above 75 nmol/L, while half (50.6%) of the participants in Brazil presented levels above this threshold. The majority (82.1%) of women living in England in comparison to 11.4% of those living in Brazil, had concentrations below the insufficiency cut off value of 50 nmol/L.

Overall (*n* = 135), serum 25(OH)D concentration was negatively correlated with age (*r* = −0.282, *p* = 0.001) and waist circumference (*r* = −0.361, *p* < 0.001), and there was a trend for a negative association with BMI (r = −0.169, *p* = 0.052) (Table 1). Within each country, no correlations were found between serum 25(OH)D concentration and age nor anthropometry (BMI or waist circumference) (all *p* > 0.406). Overall, women younger than 30 years of age had significantly higher mean 25(OH)D concentrations than those aged 30–44 years (64.7 ± 27.4 and 51.6 ± 27.2 nmol/L, *p* = 0.027); however, no significant differences amongst age groups were observed within each country (Table 1).

The proportion of white women in the England cohort was significantly higher (*p* = 0.012) while in the Brazil cohort there was an even distribution between white and brown (51.9% and 44.3%, respectively). With respect to skin type classification, the majority of participants classified themselves as type III and IV (63% overall, 66.1% of England and 60.8% of Brazil residents) (Table 1). There were no significant differences in mean serum 25(OH)D concentrations between ethno-race or skin type (all *p* > 0.081). Amongst England residents, those living in Southern England for more than 2 years had significantly lower 25(OH)D concentrations than those that had recently moved to the UK (less than a year) (*p* = 0.039) (Table 2).

### 3.2. Dietary Habits

Participant data by country for vitamin D intake have been previously published [22]. Overall (*n* = 119), mean habitual vitamin D dietary intake was 2.45 ± 1.91 μg/day. Vitamin D and calcium intakes were significantly higher in England residents compared to Brazil residents (*p* < 0.001 and *p* = 0.003, respectively). In total (*n* = 119), 21.8% had dietary vitamin D intakes below 1 μg/day and 100% had intakes below the IOM Recommended Daily Allowance (RDA) of 15 μg/day [23].

Those consuming eggs more than five times per week had significantly higher 25(OH)D concentrations (77.5 ± 30.5 nmol/L) than those consuming eggs once a week or less (47.1 ± 23.1 nmol/L, *p* = 0.001 and 51.4 ± 23.3 nmol/L, *p* = 0.014, respectively). There was also a difference in mean 25(OH)D concentration according to frequency of milk consumption, although post-hoc tests did not identify which groups differed significantly (*p* = 0.009). The same was observed for oily fish consumption within the overall sample, and a clearer difference was observed within England participants, with those consuming fish 2–5 times per week having significantly higher concentrations (48.4 ± 21.2 nmol/L) compared to those consuming it less than once a week (31.6 ± 16.2 nmol/L, *p* = 0.034). There were no significant differences according to frequency of liver consumption or level of supplement intake within the last year (Table 2).

### 3.3. Sun Exposure Behaviour

The patterns of all sunlight-related behaviour reported by the life-style questionnaire were significantly different between women living in England and women living in Brazil (*p* ≤ 0.05), except for sunscreen use in general and sun protection factor (SPF) during holidays (*p* > 0.05) (Table 3).

Overall and within just the Brazil participants, there were significant differences in serum 25(OH)D concentrations according to the number of body parts exposed (both *p* < 0.04). Amongst Brazil participants, those reporting the usual exposure of hands and face + arms and/or legs had significantly higher 25(OH)D concentrations than those exposing their hands and face only (78.5 ± 21.8 and 62.2 ± 20.0 nmol/L, respectively; *p* = 0.029).

In total, around 70% if participants reported habitual use of sunscreen, although for non-holiday use those living in England were most likely to report SPF of 15 or 20 (52.3%) compared to 95.4% of those living in Brazil who reported using SPF of 30, 40 or over (*p* = 0.003). England residents were more likely than Brazil to report natural sunbathing (41.1% and 25.3%, respectively, *p* = 0.05), whilst only 3 women in England, and none in Brazil, reported having ever used an artificial sunbed. Self-reported sunbathers were most likely to report higher SPF use on holiday (60% reported using 30, 40 or more compared to only 9% using SPF 15 or 20). There were no significant differences in 25(OH)D concentrations between sunscreen users and non-users. However, overall, those reporting using a SPF 15 sunscreen during holidays had significantly lower levels than those reporting the use of SPF ≥ 40 (34.1 ± 16.6 and 62.0 ± 29.1, respectively; *p* = 0.034).

### 3.4. Individual UVB Radiation Levels

**Figure 3:**
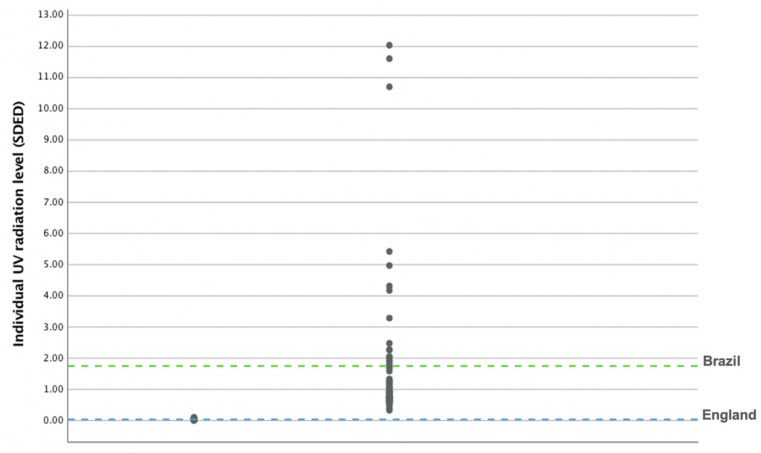
Daily individual Ultraviolet B radiation levels for women living in England (*n* = 46) and women living in Brazil (*n* = 69). Hashed lines represent mean daily individual UVB radiation level for women living in England (measured between October to March) and Brazil (measured between June to September).

The average daily individual UVB radiation levels at the beginning of winter are shown in Figure 3, labelled by country of residence. Values are expressed in units of standard erythema dose (SED) [18]. Individual UVB radiation levels differed significantly between the two countries with concentrations ranging from 0.0031 to 0.0984 SED for England residents and from 0.3283 to 12.0393 SED for Brazil residents (mean values 0.035 ± 0.026 and 1.75 ± 2.32 SED, respectively *p* < 0.001). All England dwelling participants recorded daily exposure levels of less than 1 SED compared to around half of those living in Brazil (53.6%).

Overall, vitamin D status was associated with individual UVB radiation (Table 4), with women presenting serum 25(OH)D concentrations above 75 nmol/L having significantly higher mean UVB radiation (2.26 ± 3.04 SED) than those with deficient (0.02 ± 0.01 SED), insufficient (0.25 ± 0.43 SED) or suboptimal (0.98 ± 1.00 SED) status (*p* < 0.001). Within those living in Brazil there was also a significant association (*p* < 0.040), with those in the higher vitamin D status groups presenting higher UVB radiation. There were no differences in women living in England, in which a mean SED below 0.04 was observed for all vitamin D status groups.

**Figure 4:**
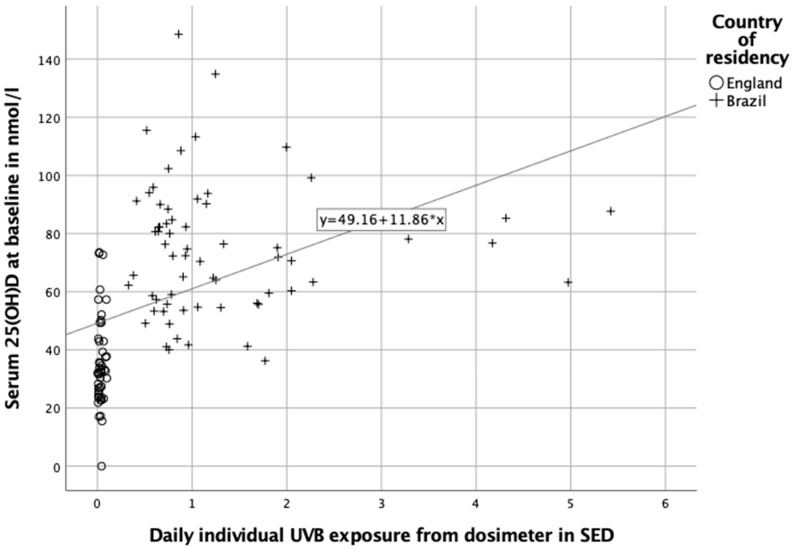
Relationship between baseline serum 25(OH)D concentration and baseline individual daily sunlight exposure level in participants with daily individual UVB exposure levels below 10 SED (*n* = 112).

Overall, daily individual UVB radiation level showed a strong significant positive correlation with serum 25(OH)D concentrations (*n* = 112, *r* = 0.673, *p* < 0.001; with *n* = 3 outliers removed from analysis due to daily SED > 10) (Figure 4) and remained statistically significant after controlling for vitamin D intake (dietary intake), age and BMI (*r* = 0.669, *p* < 0.001). In this linear model (Figure 4), a daily exposure of 0.07 SED and 2.2 SED predicted a serum 25(OH)D concentration of 50 nmol/L and 75 nmol/L, respectively.

### 3.5. Prediction of Circulating 25(OH)D Concentrations: Mathematical Modeling

Preliminary analyses ensured no violation of normality, linearity, generalizability (sample size), multicollinearity and homoscedasticity. Due to the differences in mean age and anthropometric measures and the significant correlations with serum 25(OH)D, a hierarchical multiple regression analysis was used to investigate the ability of daily individual UVB radiation levels (SED) to predict 25(OH)D concentrations (nmol/L), after controlling for the influence of age, weight and BMI.

Age and BMI were entered at Step 1, explaining 7.7% of the variance in 25(OH)D concentrations. After entry of daily individual sunlight exposure level at step 2, the total variance explained by the model as a whole was 46.5%. The added UVB radiation measure explained an additional 38.8% of the variance 25(OH)D concentrations, after controlling for the influence of age and BMI, (F (3, 111) = 32.16, *p* < 0.001). In the final model, only UVB radiation made a unique statistically significant contribution to the prediction of 25(OH)D concentrations. According to the slope coefficient for daily individual UVB radiation levels, 25(OH)D concentration increased by 20.2 nmol/L for each extra SED of UVB radiation, regardless of age and BMI (*p* < 0.001).

## 4. Discussion

Individual daily UVB radiation levels were strongly and positively correlated with serum vitamin D concentrations. Moreover, 38.8% of the total variance in 25(OH)D concentrations was explained uniquely by daily individual UVB radiation, after controlling for the influence of age and BMI. Mean serum 25(OH)D concentration of Brazilian women living in England was significantly lower than those living in Brazil. If the threshold of 75 nmol/L recommended by the Endocrine Society [16] is applied, suboptimal status was universal amongst England residents and affected half (49.3%) of the women living in Brazil.

Although significant differences in serum vitamin D concentrations between the two countries were expected, it is no less remarkable that concentrations in ethnically identical adult women ranged from 5.0 to 73.5 nmol/L for participants living in England and from 36.2 to 148.6 nmol/L for those living in Brazil. Similar serum concentrations to those observed in Brazil have been previously reported in Africa, ranging from 70 to 170 nmol/L [12], and amongst high UVB-exposure individuals (tanners, surfers, and outdoor workers, ≤162 nmol/L) [12].

The prevalence of deficiency found in this study for women living in England (25%) was similar to the yearly average of 21.7% deficiency reported for UK Caucasian adult women [14]. Brazilian women who had been living in England for more than 2 years had lower 25(OH)D concentrations than their Brazilian peers with shorter residency, indicating a worsening of status over time. Amongst the women in this study living in Brazil, there were no records of vitamin D deficiency and just less than half of the sample had insufficient or sub optimal levels. Studies conducted previously in Brazil have observed a high prevalence of insufficiency (ranging from 28–38%) and sub-optimal status (ranging from 43–81%) [24,25,26,27]. To our knowledge, our study was the first to investigate vitamin D status in the State of Goiás, located in mid-west Brazil, and the higher prevalence reported previously (in samples from southern or northern Brazil) may reflect regional and cultural differences, i.e., variations in season patterns and climate, and cultural habits with regards to sun exposure behaviour and food intake.

It was estimated in this cohort, that each extra SED of UVB radiation (a safe limit of daily UVB radiation level enough to produce vitamin D for skin types I-IV [28]), would increase 25(OH)D concentration by 20.2 nmol/L, independent of age and BMI. Furthermore, a daily individual UVB radiation of around 2 SED would be required to maintain serum 25(OH)D at 75 nmol/L. It is surprising that such a high proportion of participants in Brazil presented relatively low individual daily exposure levels (53.6% < 1SED), considering the high minimum winter UVB index in Brazil of 8. However, this may reflect known influential factors such as behaviour towards sunlight exposure, pollution, weather variations, sun avoidance due to concerns regarding skin damage and time spent indoors (i.e., work, physical activity and commuting inside vehicles) [29].

The importance of direct skin exposure was also shown, since those women living in Brazil who exposed more body parts had a better Vitamin D status. In fact, a recent study conducted in Ireland, with 5138 community-dwelling participants aged >60 years and blood sampling throughout the year, showed that individuals who avoided sun exposure were at higher risk of deficiency (<40 nmol/L), whilst those who reported enjoying sun exposure tended to be vitamin D sufficient (≥50 nmol/L). Moreover, UVB dose and sunshine enjoyment seemed to improve prediction of vitamin D deficiency in individuals not taking supplements [30]. Such observations build on the affirmation that individual UVB radiation level is better determined by individual behaviour towards sunlight rather than estimated local UVB radiation availability and so recommendations need to help individuals to optimize their own sun exposure to balance safety and vitamin D production.

Additionally, although there were no differences in mean 25(OH)D concentrations between sunscreen users and non-users, those reporting using a SPF 15 sunscreen during holidays had significantly lower Vitamin D levels than those reporting the use of SPF of ≥40. This suggests that higher factor use may be a marker for higher overall exposure and greater likelihood of sunbathing. In fact, it was observed in this study that self-reported sunbathers were more likely to use higher factor sunscreen on holiday, supporting this behavioural association. A similar study conducted in Australia did not observed any statistically significant association between the 25(OH)D concentrations and the use of sunscreens, but also reported that participants who used sunscreen presented some of the highest 25(OH)D concentrations. The authors hypothesized that the use of sunscreens is likely to be an indicator of increased sun exposure in general [10]. Such observations reinforce the importance of considering habitual behaviour towards sunlight and how it can affect 25(OH)D cutaneous production in the skin when determining vitamin D and sunlight exposure recommendations for different populations. From a holistic point of view, taking high factor sunscreen alone as an indicator of lower UVB radiation reaching the skin, for instance, could potentially underestimate individual exposure UVB radiation level if higher SPF is also a marker for greater length of time spent in the sun.

The influence of skin pigmentation has also been well observed in studies showing poorer vitamin D status in dark-skinned compared to lighter-skinned individuals, with higher amounts of UVB required in pigmented skins to achieve adequate 25(OH)D concentrations. In the present study, no associations were found between 25(OH)D concentrations and self-declared ethno-race or skin type. The reason for this is likely due to the potential inconsistency in self-declared ethno-race and skin colour in the Brazilian population because of subjective definitions and cultural influence on ethnic identification. This was indeed observed in this study where 63% of participants identified themselves as white and 33.3% as brown while, conversely, 31.1% classified themselves as type I and II (white) and 63% as type III and IV (light and moderate brown). This inconsistency suggests that simple classifications of ethnicity and skin colour to investigate the effect of skin pigmentation on vitamin D status might not be appropriate for some populations or countries. Other more objective methods such as measures of melanin density via spectral reflectance of the skin [10] or classification by a trained researcher based on observed skin type characteristics may be better options in certain populations.

There are still very few studies that have investigated the effects of UVB exposure in vivo in South America, and specifically in Brazil, which has led to formulation of recommendations based on data derived from studies conducted mainly in the USA and Europe, where the UVB availability and the sun exposure habits are considerably different from those observed in low latitude countries. The strength of this cross-sectional analysis is the directly comparable data on serum 25(OH)D concentrations and daily individual UVB radiation measurement that represents personal and habitual solar radiation in real-life scenarios. This study addresses key knowledge gaps with two parallel studies, using identical methodologies to examine same sex and ethnicity individuals (minimizing confounding due to cultural habits and skin pigmentation), directly comparing individual UVB radiation levels and habitual behaviour towards sunlight between high and low latitudes. Further strengths of the present study include serum 25(OH)D measurement via liquid chromatography–mass spectrometry, the gold-standard method for assessing vitamin D status and data collection during the same season in both countries. Furthermore, to date, this is also the first study to measure habitual UBV radiation levels using a personal UVB dosimeter in Brazil and the first study to show the strong correlation of individual levels with vitamin D serum concentrations in the Brazilian population.

It is important to note that these findings may not be generalizable to other groups such as men, children, adolescents and pregnant or older women and other ethnic groups with different characteristics, habits or culture. Participants in this study had a generally healthy BMI and therefore, findings may not reflect populations with overweight or obesity due to the known influence of adiposity on vitamin D status. The study was conducted during wintertime in England in order to achieve a minimal sun exposure so this cohort would represent the minimal habitual UVB exposure in comparison to a high (but not extreme) exposure in Brazil. Additionally, the study was conducted in Brazil also during wintertime so that we could minimize the extreme radiation and boosted sunlight exposure habits during summer. Alternatively, the same study could have been done during summertime in both latitudes in order to compare differences in the influence of sun exposure when sunlight availability is at its highest.

## 5. Conclusions

The prevalence of vitamin D deficiency and insufficiency was extremely high in adult Brazilian women residing in southern England. This study has highlighted the strong positive association between vitamin D serum 25(OH)D concentrations and individual UVB radiation. Given the perhaps previously underappreciated variation in individual UVB radiation levels this study highlights the importance of measuring rather than assuming individual exposure. Vitamin D deficiency or inadequacy, strongly associated with low individual UVB radiation levels, could put these women at a greater risk of poor bone health at the end of winter, particularly in England. Further work should focus on extending the sample to include a wider demographic range, including more overweight individuals, males, and other ethnic and age groups in different latitudes.

## Figures and Tables

**Table 1:** Socio-demographic characteristics of adult Brazilian women overall and by country of residence and associations with serum 25(OH)D concentrations (*n* = 135) ^1^**^.^**

					All		England		Brazil	
	All	England (*n* = 56)	Brazil (*n* = 79)	*p*	25(OH)D in nmol/L	*p^2^*	25(OH)D in nmol/L	*p^2^*	25(OH)D in nmol/L	*p^2^*
Age (years)	31.39 ± 8.7	35.55 ± 9.0	28.43 ± 7.1	<0.001 ^2^						
<30	71 (53%)	18 (32%)	53 (67%)		64.7 ± 27.4 ^a^	0.030	13.2 ± 3.1	0.483	74.1 ± 24.4	0.761
30–44	48 (35%)	29 (52%)	19 (24%)		51.6 ± 27.2 ^a^		15.7 ± 2.9		78.2 ± 18.0	
>44	16 (12%)	9 (16%)	7 (9%)		54.8 ± 21.1		14.9 ± 4.9		72.5 ± 12.9	
Ethno-race [*n* (%)] ^R1^										
White	85 (63%)	44 (78.6%)	41 (51.9%)	0.012 ^3^	55.6± 25.8	0.207	37.4 ± 15.9	0.529	75.2 ± 19.3	0.422
Black	3 (2.2%)	1 (1.8%)	2 (2.5%)		43.1 ± 15.3		25.7		51.8 ± 4.1	
Brown	45 (33.3%)	10 (17.9%)	35 (44.3)		66.3 ± 29.5		30.9 ± 8.2		76.4 ± 25.2	
Yellow	1 (0.7%)	0	1 (1.3%)		59.5		N/A		59.5	
Indigenous	1 (0.7%)	1 (1.8%)	0		42.9		42.9		N/A	
Skin type [*n* (%)] ^R2^										
Type I and II	42 (31.1%)	17 (30.4%)	25 (31.6%)	0.589 ^3^	61.0 ± 24.9	0.705	37.2 ± 12.5	0.927	77.3 ± 16.6	0.812
Type III and IV	85 (63%)	37 (66.1%)	48 (60.8%)		57.4 ± 28.9		35.7 ± 16.0		74.9 ± 25.5	
Type V and VI	8 (5.9%)	2 (3.6)	6 (7.6)		63.0 ± 21.0		33.9 ± 13.9		72.7 ± 11.4	
Years in England [*n* (%)]							51.0 ± 14.9 ^a^	0.039		
<1 year	N/A	6 (10.73%)	N/A	N/A			37.4 ± 11.3			
1–2 years		10 (17.85%)					33.5 ± 14.6 ^a^			
>2 years		40 (71.42%)								

^1^ Values are mean ± Standard deviation or *n* (%). Statistical analysis: ^2^ One-way ANOVA with post-hoc Tukey’s test. Values in same column with same superscript letters are significantly different (*p* < 0.05). ^3^ Pearson Chi Squares. References: ^R1^ Official ethno-racial categories for the Brazilian population by the Brazilian Institute of Geography and Statistics (Brown: mixed; Yellow: Japanese-descendant; Indigenous: native Indian) [20] ^R2^ The Fitzpatrick Classification Scale for Skin Types [21]. N/A: Not Applicable.

**Table 2:** Lifestyle characteristics of adult Brazilian women overall and by country of residence (*n* = 135) ^1^**^.^**

	All	England(*n* = 56)	Brazil(*n* = 79)	*p* ^2^	All		England		Brazil	
					25(OH)D in nmol/L	*p* ^3^	25(OH)D in nmol/L	*p* ^3^	25(OH)D in nmol/L	*p* ^3^
Alcohol Consumption										
No	58 (43%)	34 (60.7%)	36 (45.6%)	0.467	57.57 ± 25.6	0.674	37.9 ± 16.9	0.253	76.9 ± 24.0	0.396
Yes	77 (57%)	22 (39.3%)	43 (54.4%)		59.7 ± 28.7		33.2 ± 11.1		72.6 ± 19.6	
Smoke										
No	126 (93.3%)	5 (8.9%)	75 (94.9%)	0.375	60.2 ± 22.6	0.861	43.2 ± 10.1	0.266	81.6 ± 11.6	0.545
Yes	9 (6.7%)	51 (91.1%)	4 (5.1%)		58.6 ± 27.8		35 ± 15.2		74.6 ± 22.6	
Milk consumption/day										
Never	39 (28.7%)	13 (23.2%)	28 (35.5%)	0.001	65.9 ± 30.11	0.009	37.4 ± 16.7	0.928	78.6 ± 25.7	0.613
Less than 1 mug	31 (23%)	17 (30.4%)	14 (17.7%)		51.84 ± 23.5		37.1 ± 16.2		68.9 ± 18.9	
1 mug (280 mL)	38 (28.1)	9 (16.1%)	29 (36.7%)		65.0 ± 26.5		33.5 ± 11.7		74.7 ± 21.8	
2 mugs (560 mL)	21 (15.6%)	13 (21.3%)	8 (10.1%)		49.3 ± 24.7		34.7 ± 15.7		73.1 ± 16.7	
More than 2 mugs	4 (3%)	4 (7.1%)	0		31.3 ± 6.74		31.3 ± 6.7		N/A	
Egg consumption/week										
Never	4 (2.9%)	1 (1.8%)	3 (3.8%)	0.203	62.5 ± 15.7	0.002	52.1	0.091	67.8 ± 18.2	0.095
Less than once	21 (15.6%)	9 (16.1%)	12 (15.2%)		51.4 ± 23.3 ^a^		30.2 ± 14.3		66.8 ± 16.1	
Once a week	29 (21.5%)	17 (30.4%)	12 (15.2%)		47.1 ± 23.1 ^b^		30.6 ± 9.1		70.4 ± 15.0	
2–5 times	61 (45.2%)	24 (42.9%)	37 (46.8%)		60.3 ± 26.6		39.5 ± 15.7		73.9 ± 23.4	
>5 times	20 (14.8%)	5 (8.9%)	15 (19%)		77.5 ± 30.5 ^a,b^		44.9 ± 21.5		88.3 ± 25.1	
Oily fish consumption/week										
Never	31 (22.9%)	10 (17.9%)	20 (25.3%)	0.005	56.1 ± 23.3	0.047	31.9 ± 12.1	0.034	68.1 ± 17.4	0.215
Less than once	58 (43%)	17 (30.4%)	41 (51.9%)		64.6 ± 30.5		31.6 ± 16.2 ^a^		78.4 ± 23.4	
Once	33 (24.4%)	21 (37.5%)	12 (15.2%)		51.2 ± 24.2		37.13 ± 8.7		75.8 ± 22.9	
2–5 times	12 (8.9%)	8 (14.3%)	4 (5.1%)		52.7 ± 20.8		48.4 ± 21.2 ^a^		61.1 ± 20.0	
>5 times	1 (0.7%)	0	1 (1.3%)		109.7		N/A		109.7	
Liver consumption/week										
Never	100 (77%)	45(81.8%)	55 (70%)	0.520	59.1 ± 28.1	0.645	36.9 ± 15.5	0.594	76.9 ± 22.8	0.483
Less than once	28 (20.7%)	9 (16%)	19 (24%)		55.9 ± 25.5		30.3 ± 10.1		68.9 ± 21.2	
Once	2 (1.5%)	1 (2%)	1 (3%)		55.8 ± 35.1		31.0		80.7	
2–5 times	1 (0.7%)	0	1 (3%)		84.7		N/A		84.7	
>5 times	0	0	0		n/a		n/a		n/a	
Supplement use (within the last year)										
None	86 (70%)	32 (57.2%)	54 (68.4%)	0.002	61.4 ± 28.3	0.189	36.3 ± 17.2	0.866	75.5 ± 23.1	0.382
Vitamin D	15 (11.1%)	3 (5.4%)	12 (15.2%)		64.5 ± 22.1		42.9 ± 7.2		68.9 ± 21.3	
Fish/fish liver oil	3 (2.2%)	2 (3.6%)	1 (1.3%)		45.4 ± 30.5		28.2 ± 9.0		80.0	
Fish/liver oil with vit. D	14 (10.4%)	5 (8.9%)	9 (11.4%)		55.2 ± 22.6		30.4 ± 5.3		69.0 ± 15.1	
Multivitamin with vit. D	13 (9.6%)	12 (21.4%)	1 (1.3%)		41.9 ± 25.3		35.9 ± 14.0		113.3	
Calcium with vit. D	4 (3%)	2 (3.6%)	2 (2.5%)		64.2 ± 34.3		37.7 ± 2.1		90.8 ± 26.72	

^1^ Values are *n* (%). ^2^ Statistical analysis: Chi Squares. ^3^ One-way ANOVA with post-hoc Tukey’s test or *t*-test. Values in same column with same superscript letters are significantly different (*p* < 0.05). N/A: not applicable.

**Table 3:** Sun-exposure behaviours of adult Brazilian women overall and by country of residence (*n* = 135) ^1^.

	All	England (*n* = 56)	Brazil (*n* = 79)	*p* ^2^
Body parts exposed				
Face only	1 (0.7%)	1 (1.8%)	0	<0.001
Hands and face	48 (35.6%)	33 (58.9%)	15 (19%)	
Hands/face + arms and/or legs	71 (52.6%)	15 (26.8%)	56 (70.9%)	
Hands/face + arms/legs + torso	15 (11.1%)	7 (12.5%)	8 (10.1%)	
Sunscreen use				
No	41 (30.4%)	14 (25%)	27 (34.2%)	0.253
Yes	94 (69.6%)	42 (75%)	52 (65.8%)	
SPF at home ^§^				
15	11 (11.7%)	10 (23.8%)	1 (2.27%)	0.003
20	14 (14.8%)	12 (28.5%)	1 (2.27%)	
30	30 (31.9%)	6 (14.2%)	23 (54.5%)	
40 or over	32 (34.0%)	14 (32.5%)	18 (40.9%)	
Missing	7 (7.4%)	0	7	
SPF on holidays ^§^				
15	8 (8.4%)	7 (16.7%)	1 (0.19%)	0.06
20	3 (3.2)	2 (4.7%)	1 (0.19%)	
30	34 (35.8%)	17 (40.6%)	17 (32.6%)	
40 or over	39 (41%)	15 (35.8%)	24 (46.1%)	
Missing	11 (11.6%)	1 (2.1%)	9 (21.4%)	
Natural sunbathing habit				
No	92 (68.1%)	33 (58.9%)	59 (74.7%)	0.05
Yes	43 (31.9%)	23 (41.1%)	20 (25.3%)	
Artificial sunbed use				
No	131 (97%)	52 (92.9%)	79 (100%)	0.036
Yes	3 (2.2%)	3 (5.4%)	0	
Missing	1 (0.8%)	1 (1.7%)	0	

^1^ Values are *n* (%).^2^ Statistical analysis: Pearson Chi Squares. ^§^ Amongst participants who said “Yes” to previous item. *SPF*: Sun Protection Factor.

**Table 4:** Association between Vitamin D status and mean individual UVB radiation level ^1^.

	All			England			Brazil		
Vitamin D status	*n*	Mean ± SD	*p* ^2^	*n*	Mean ± SD	*p* ^2^	*n*	Mean ± SD	*p* ^2^
<25 nmol/L	12	0.02 ± 0.01 ^a^	<0.001	12	0.02 ± 0.018	0.666	0	N/A	0.040
25–49.9 nmol/L	34	0.25 ± 0.43 ^b^		26	0.038 ± 0.028		8	0.94 ± 0.42	
50–74.9 nmol/L	33	0.98± 1.00 ^c^		8	0.037 ± 0.027		25	1.28 ± 0.97	
>75 nmol/L	36	2.26 ± 3.04 ^a,b,c^		0	N/A		36	2.26 ± 3.04	

^1^ Values: mean ± SD. ^2^ Statistical analysis: one-way ANOVA with post-hoc Tukey’s test. Values in same column with same superscript letters are significantly different (^a^ *p* = 0.002; ^b^ *p* < 0.001; ^c^ *p* = 0.020). N/A: not applicable.
